# The Pivotal Role of the Western Diet, Hyperinsulinemia, Ectopic Fat, and Diacylglycerol-Mediated Insulin Resistance in Type 2 Diabetes

**DOI:** 10.3390/ijms26189191

**Published:** 2025-09-20

**Authors:** Joseph A. M. J. L. Janssen

**Affiliations:** Department of Internal Medicine, Erasmus Medical Center, Rotterdam, Dr. Molewaterplein 40, 3015 GD Rotterdam, The Netherlands; j.a.m.j.l.janssen@erasmusmc.nl; Tel.: +31-0612752413

**Keywords:** Western diet, overnutrition, food additives, hyperinsulinemia, insulin resistance, type 2 diabetes, obesity, bariatric surgery, membrane-bound diacylglycerol, VLCD diets, caloric restriction, ectopic fat, remission of type 2 diabetes

## Abstract

Genetic background, the “Western diet”, and environment may all contribute to hyperinsulinemia. Hyperinsulinemia can precede and cause insulin resistance. In situations of fuel overload, insulin resistance limits the amount fuel (glucose and fatty acids) entering insulin-sensitive tissues. When energy intake is chronically greater than energy expenditure, the capacity of the subcutaneous fat tissues to store fat can be overpowered. If subcutaneous fat tissues are no longer able to accommodate excess energy, there will be spillover of lipids. Excess calories will be stored as ectopic fat (triglycerides) in the liver, pancreas, and skeletal muscle. Growing evidence suggests that ectopic fat deposition directly causes insulin resistance and pancreatic beta cell dysfunction. Overnutrition and ectopic fat increase diacylglycerol (DAG) accumulation in fat cells, hepatocytes, and skeletal muscle cells. A unifying hypothesis proposes that translocated DAG into the plasma membrane induces insulin resistance in all these three cell types. In addition, ectopic fat accumulation in the pancreas induces beta-cell dysfunction. Introducing a negative energy balance by bariatric surgery or a very low-calorie diet (VLCD) reduces ectopic fat depositions from the liver and pancreas and decreases intracellular DAG content: both are effective treatments to restore insulin sensitivity, normalize metabolism, and put type 2 diabetes in remission.

## 1. Introduction

Pancreatic insulin secretion rises with increasing plasma glucose concentrations. In individuals with normal glucose tolerance and insulin receptor sensitivity, glucose homeostasis is achieved with relatively low insulin levels. Plasma glucose levels and insulin concentrations in individuals with normal glucose tolerance and impaired glucose tolerance show a positive correlation during an oral glucose tolerance test [1]. When blood glucose levels further rise into the range of overt type 2 diabetes, glucose-induced pancreatic insulin secretion may progressively decline due to exhaustion of the pancreatic beta cells [1]. Consequently, late stages of type 2 diabetes are characterized by progressive insulin deficiency and (untreated) by rising glucose concentrations. Thus, during development from normoglycemia to frank type 2 diabetes, insulin secretion follows a pattern of an inverted U: insulin concentrations initially rise, reach a peak at a certain moment, and then decrease when frank diabetes develops [2].

## 2. Fasting Hyperinsulinemia May Precede Insulin Resistance

**Figure 1:**
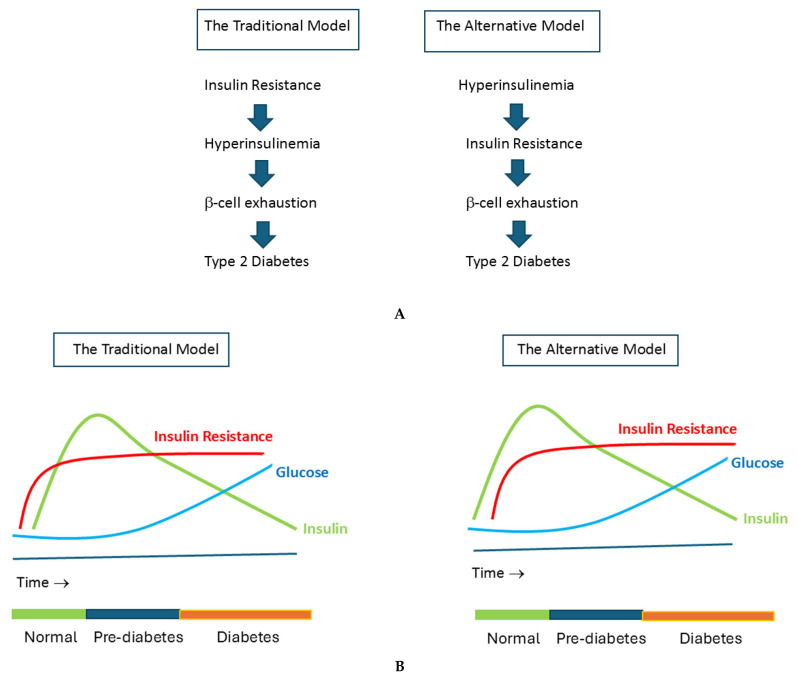
(**A**) The Traditional Model vs. The Alternative Model of the pathogenesis of type 2 diabetes. The Traditional Model posits that insulin resistance is the primary abnormality leading to hyperinsulinemia, which is followed by β-cell dysfunction. When β-cells are no longer able to sustain sufficient insulin secretion to compensate for hyperglycemia, they become exhausted and frank type 2 diabetes will develop. In the Alternative Model, chronic hypersecretion of insulin (due to genetics, overnutrition, food additives, and environmental factors) is the primary abnormality leading to hyperinsulinemia. Hyperinsulinemia initiates and sustains the development of insulin resistance (and obesity), until the β-cells fail and become exhausted and ultimately, frank type 2 diabetes develops. (**B**) In the Traditional Model, insulin resistance (1) precedes hyperinsulinemia (2), which is followed by β-cell exhaustion and finally, frank type 2 diabetes. In the Alternative Model, hypersecretion of insulin and the resulting hyperinsulinemia (1) primarily cause insulin resistance (2), which is followed by β-cell exhaustion and finally, frank type 2 diabetes. Note that in The New Model, hyperinsulinemia is already present when there is a normal glucose tolerance. Reproduced from [9].

Type 2 diabetes is a complex and multifactorial disease characterized by impaired insulin secretion and insulin resistance. Both hyperinsulinemia and insulin resistance are known risk factors for type 2 diabetes. Changes in pancreatic insulin secretion and insulin sensitivity may arise many years before the development of type 2 diabetes [3]. The term insulin resistance refers to decreased insulin receptor sensitivity. Decrease in insulin action may occur at the receptor and postreceptor level of the insulin receptor and is tissue specific [4]. In individuals with insulin resistance insulin signaling is impaired at the level of Insulin Receptor Substrate-1 (IRS-1), leading to decreased glucose transport/phosphorylation/metabolism and impaired nitric oxide synthase activation/endothelial dysfunction [5]. At the same time, insulin signaling through the Mitogen-Activated Protein Kinase (MAPK) pathway remains normally sensitive to insulin [5]. For many years, the prevailing view was that insulin resistance (that is, resistance to insulin’s role in promoting glucose uptake by muscle and fat cells) preceded and caused hyperinsulinemia. In this view, insulin resistance was the initial defect leading to the development of metabolic syndrome, hyperglycemia, and type 2 diabetes after years or even decades later [6,7,8,9] (Figure 1). If cells became resistant to insulin, the pancreas initially secreted more insulin compensating for insulin resistance in liver (glucose production) and peripheral tissues (glucose disposal in skeletal muscle and fat cells). Plasma glucose levels remained in the normal range if the pancreas was able to produce enough insulin to compensate for insulin resistance. If the pancreatic beta cells lost the ability to produce enough insulin, this led to elevated blood glucose levels, and eventually to impaired glucose tolerance and/or type 2 diabetes.

The view that insulin resistance is the principal cause of type 2 diabetes has been challenged in the last years. Insulin secretion appears to be more heritable than insulin resistance: in genome-wide association studies (GWASs), only a few loci point to insulin resistance as the primary cause of type 2 diabetes, whereas the majority of the loci identified by GWAS point towards defects in pancreatic beta-cells, specifically impacting insulin secretion [10,11,12]. Moreover, a large part of individuals with normal glucose tolerance already show hyperinsulinemia before the development of impaired glucose tolerance/obesity. For example, Ferrannini et al. found increased plasma insulin levels in subjects with normal or near normal glucose tolerance [13]. It is inexplicable how insulin resistance in subjects with normal glucose tolerance could be responsible for increased insulin secretion when blood glucose concentrations are still normal and this is another argument against a primary role for insulin resistance-mediated hyperinsulinemia [1]. These findings raise the distinct possibility that a pancreatic beta-cell defect in insulin secretion may be the primary driver in the development of insulin resistance/type 2 diabetes. Based on these latter considerations an alternative model has been proposed. In this alternative model, hyperinsulinemia is considered the primary defect in which hyperinsulinemia precedes and directly causes insulin resistance (Figure 1). In the long term, this may potentially lead to pancreatic beta cell exhaustion and type 2 diabetes [1,14,15] (Figure 1).

The development of insulin resistance has been proposed to be a defense mechanism of the body protecting insulin-sensitive tissues against fuel overload and fuel toxicity [16,17] (see also below).

It is widely accepted that hypersecretion of a hormone can lead to desensitization of its receptor and signaling pathway at the cellular level, primarily through downregulation of the receptor [18]. This concept also applies to hyperinsulinemia and the insulin receptor [19]. Continuous and prolonged elevation of plasma insulin levels induce insulin receptor resistance through multiple mechanisms at the insulin receptor and postreceptor level [9]. Hyperinsulinemia induces downregulation of the number of insulin receptors at the cellular surface of the target tissues by promoting intracellular internalization as well as degradation of insulin receptors [20]. Hyperinsulinemia can also downregulate signaling of the insulin receptor at the level of effector molecules, disrupting phosphorylation of Insulin Receptor Substrate-1 (IRS-1), Phosphatidylinositol 3-kinase (PI3K), and Protein Kinase B (Akt) [18].

Support for a causal role of hyperinsulinemia in the development of insulin resistance is provided by a prospective study in Pima Indians demonstrating that pancreatic hypersecretion of insulin is the first defect that occurs prior to insulin resistance [21]. In 1168 adults and adolescents, primary insulin hypersecretion, independent of insulin resistance, was associated with a worse clinical and metabolic phenotype and this predicted deterioration of glucose control over time [22]. In another study, hyperinsulinemia was present in 221 apparently healthy young (18–35 years), normoglycemic non-obese individuals [23]. The hyperinsulinemia in this study was the consequence of a combined effect of an increased insulin secretion and reduced insulin clearance [23]. Many other human prospective studies support the hypothesis that basal hyperinsulinemia precedes insulin resistance [9,14]. Further support for a primary role of hyperinsulinemia in the development of insulin resistance comes from a longitudinal study of Rhesus monkeys with type 2 diabetes [24]. In these monkeys, a 10-fold increase in basal (fasting) plasma insulin levels and a five-fold increase in insulin response to glucose were observed in the earliest stages of type 2 diabetes [24]. Moreover, these changes occurred independent of degree of obesity and before the development of hyperglycemia [24]. Thus, this observation suggests that development of type 2 diabetes starts with increased insulin secretion [24].

When non-diabetic subjects with insulin resistance but without hyperinsulinemia were compared with non-diabetic subjects with hyperinsulinemia without insulin resistance, significant differences were found in the respective clinical phenotypes, suggesting that subjects with primary hyperinsulinemia and subjects with primary insulin resistance carry distinct pathogenic potential in terms of the components of the insulin resistance syndrome [25].

Discussions about the “egg and chicken” relationship between hyperinsulinemia and insulin resistance have important clinical implications [26]. When hyperinsulinemia is a compensatory response to insulin resistance as proposed in the traditional view, an increase in plasma insulin concentrations and insulin therapy may overcome insulin resistance and help to achieve a better metabolic control and long-term outcome [26]. However, when hyperinsulinemia is the primary defect that triggers insulin resistance, as proposed in the alternative model, therapeutic strategies should primarily focus on reducing plasma insulin concentrations to improve metabolic control and long-term outcomes [26].

## 3. Fasting Hyperinsulinemia Is a Premature Sign of Type 2 Diabetes

**Figure 2:**
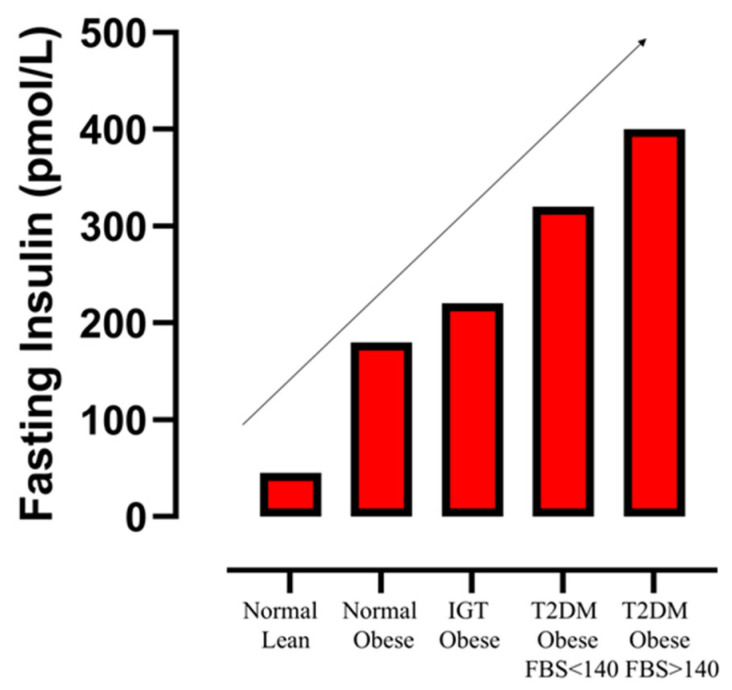
Mean fasting plasma insulin levels progressively increase during progression from normal glucose tolerance to type 2 diabetes. Mean fasting levels in lean individuals with normal glucose tolerance (Normal Lean), in individuals with normal glucose tolerance and obesity (Normal Obese), in individuals with impaired glucose tolerance and obesity (IGT Obese), individuals with type 2 diabetes, obesity, and fasting blood glucose levels < 140 mg/dL (7.8 mmol/L) (T2DM Obese < 140), and individuals with type 2 diabetes, obesity, and fasting blood glucose levels > 140 mg/Dl (7.8 mmol/L) (T2DM Obese > 140). Note: fasting plasma insulin concentrations continue to rise from normoglycemia to impaired glucose tolerance to type 2 diabetes. In the early phases of development of type 2 diabetes, fasting insulin levels in people with type 2 diabetes with fasting blood glucose levels >140 mg/dL (7.8 mmol/L) were 9-fold higher than in lean people with normal fasting glucose levels. Modified from [1].

Pories et al. found (in a cross-sectional study) that during progression from normal glucose tolerance to type 2 diabetes, maximum fasting plasma insulin concentrations in people with type 2 diabetes were still twice as high as in euglycemic lean individuals, despite the fact that peak insulin secretion in the former individuals was decreased after an oral glucose load [27]. Even more important was the observation that fasting plasma insulin concentrations continued to rise from normoglycemia to impaired glucose tolerance to type 2 diabetes [1,27] (Figure 2). Consequently, in the early phases of development of type 2 diabetes, fasting insulin levels in people with type 2 diabetes with fasting blood glucose levels > 140 mg/dL (7.8 mmol/L) were nine-fold higher than in lean people with normal fasting glucose levels (8) (Figure 2). Many other longitudinal studies have also demonstrated that in subjects with normal glucose tolerance, basal/fasting hyperinsulinemia (independent of insulin resistance) is a premature feature predicting the development of type 2 diabetes during follow-up [21,28,29,30,31,32]. Moreover, fasting insulin is associated with diurnal insulin exposure [33]. In addition, in many populations with high prevalence rates of type 2 diabetes (such as Native Americans, Pacific Islanders, African Americans, and Mexican Americans), relatively high mean fasting/basal plasma insulin levels compared to those in Caucasians are already present before developing dysglycemia [21,30,34,35,36,37].

All these data support the hypothesis that fasting/basal hyperinsulinemia precedes development of both insulin resistance and type 2 diabetes.

## 4. What Is the Cause of Hyperinsulinemia?

Hyperinsulinemia can be caused by increased pancreatic insulin secretion in the fasting state or postprandially after ingestion of carbohydrates and/or free fatty acids [38]. Hyperinsulinemia may be further caused by decreased hepatic insulin clearance [39]. Thus, both increased pancreatic insulin secretion and decreased hepatic insulin extraction may cause hyperinsulinemia [39]. Hyperinsulinemia may be further caused by a compensatory (secondary) response to insulin receptor resistance [38].

**Figure 3:**
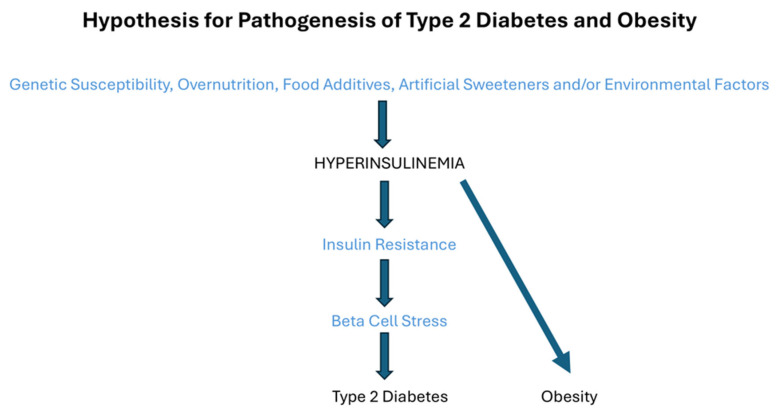
Hypothesis for pathogenesis type 2 diabetes and obesity. In 2011 Barbara Corkey proposed in her Banting Lecture in 2011 that overnutrition (excess nutrient ingestion), food additives, artificial sweeteners, and environmental factors induce hyperinsulinemia superimposed on a susceptible genetic background of basal insulin levels [15]. Hyperinsulinemia precedes and causes insulin resistance and obesity. The model also hypothesizes that in the long- term hyperinsulinemia-mediated insulin resistance may be contributory or a major cause of type 2 diabetes (see text for details).

In 2011, Barbara Corkey proposed in her Banting lecture that long-term overnutrition (excess nutrient ingestion), food additives, artificial sweeteners, and environmental factors induce hyperinsulinemia superimposed on a susceptible genetic background of basal insulin levels, and she suggested that this combination of factors may be major cause of the current increased prevalence of type 2 diabetes and obesity [15] (Figure 3). Thus, in this concept genetic susceptibility, consumption of the “modern” Western diet, and environment, may all cause and contribute to (primary) hyperinsulinemia by enhancing pancreatic insulin secretion, decreasing insulin pulses, and/or reducing hepatic insulin clearance [9] (Figure 3). In favor of an important role for environmental factors in the development of type 2 diabetes, is the much lower prevalence of type 2 diabetes in the Pima Indians in Mexico than in the U.S [40]. This suggests that in populations who share a common genetic background and possess a comparable genetic predisposition to type 2 diabetes, type 2 diabetes is determined mostly by differences in lifestyle (including diet and physical (in)activity) [40]. In addition, this observation provides evidence that changes in lifestyle associated with Westernization play a major role in increasing prevalence of type 2 diabetes and obesity [40]. Twin studies provided evidence that heritability for fasting insulin and for 30-min insulin after an oral glucose load was 37–54% and 47–57%, respectively [41]. More than 400 genetic variants have been identified which are implicated in the risk of type 2 diabetes [42]. Many of these genetic variants modulate type 2 diabetes risk through direct or indirect effects on insulin secretion [43]. In addition, genome-wide association studies (GWASs) in large multiethnic population-based cohorts identified many genetic alleles associated with increased fasting serum insulin levels [44]. There is ample evidence that the pattern of pancreatic insulin secretion also determines hepatic insulin clearance: the liver preferentially extracts insulin when it is delivered to the liver in pulses [45]. Thus, (loss of) pulsatile pancreatic insulin secretion dictates both hepatic (directly) as well as extra-hepatic (indirectly) insulin delivery [45]. Hepatic insulin clearance may change within days to altered dietary energy intake and thereby function as a major regulator of systemic (=posthepatic) insulin concentrations [46]. Hepatic insulin clearance shows wide variance among individuals and ethnic backgrounds [47]. For example, mean hepatic insulin clearance is 67% lower in African Americans than European Americans [47]. Hepatic insulin clearance is a highly heritable trait, and several loci have been identified that harbor genes regulating insulin clearance [48]. Some years ago, Bergman et al. hypothesized that low (ered) hepatic clearance was the primary abnormality in the development of type 2 diabetes [47,49]. They proposed that peripheral hyperinsulinemia in the prediabetic situation is caused by reduced hepatic insulin clearance rather than by pancreatic insulin overproduction [47]. However, lowered hepatic clearance is currently not considered as the major cause for development of hyperinsulinemia and type 2 diabetes.

## 5. Hyperinsulinemia and Obesity

A major effect of insulin (hyperinsulinemia) is promoting lipogenesis by stimulating fatty acid uptake and triglyceride synthesis in fat cells [50,51,52]. Insulin also inhibits lipolysis [50,53]. Thus, when there is full sensitivity to insulin-mediated lipogenesis and anti-lipolysis, hyperinsulinemia and intake of excess calories will promote development and maintenance of fat mass [54]. As noted earlier, hyperinsulinemia can be present in apparently healthy and non-obese subjects without significant insulin resistance, suggesting that hyperinsulinemia may be the initial trigger which subsequently generates lipogenesis, insulin resistance and obesity [55]. In a prospective study, Trico et al. found that insulin hypersecretion in adolescents with obesity, but without diabetes, was associated with increased triglyceride synthesis in subcutaneous adipose tissue, adipocyte hypertrophy, ectopic fat accumulation, and greater body fat gain (≈2% of total fat mass) during the 2-year observation period [56]. Thus, this study provides evidence that insulin hypersecretion in an obesogenic context can promote obesity [57]. Moreover, overnutrition in utero may cause fasting hyperinsulinemia during childhood in the absence of overweight/obesity [58]. In addition, higher fasting insulin levels during childhood have been associated with the development of insulin resistance, abnormal glucose tolerance, and obesity in the following 6 years [58]. Moreover, epidemiological studies in young children (without insulin resistance) found that hyperinsulinemia is a predictor for body weight gain and obesity later in life [34,59].

Genetic studies are often used to provide better insights into cause-and-effects and the role of specific components in the pathogenesis of a condition/disease. A bi-directional Mendelian Randomization study recently examined the relationship between insulin secretion and BMI in humans and found that genetically determined insulin secretion strongly predicted BMI, whereas, in contrast, genetically determined BMI did not predict insulin secretion [60]. In another study, insulin promotor gene variants were associated with insulin hypersecretion and predicted weight gain during adolescence [61]. These findings are consistent with data in animals demonstrating that downregulation of insulin production can reverse or prevent body fat accumulation, including ectopic fat [62]. All these data suggest that hyperinsulinemia per se—independent from insulin resistance— may be an important and primary driver of diet-induced obesity.

## 6. The Modulation of Food Intake by Central Insulin Action and Central Insulin Resistance

Insulin from the blood can penetrate into the cerebrospinal fluid (CSF) at a slow rate and the insulin levels within the CSF are an integral over time of the levels in the blood [63]. While relatively high insulin levels after food intake stimulate energy storage in the liver, fat, and muscle, they simultaneously decrease food intake in the central nervous system (CNS) [64]. Basal insulin level is an important determinant of insulin sensitivity [65]. Chronic high plasma insulin levels may lead to the desensitization of the insulin receptors and dysregulation of insulin signaling in insulin receptor-expressing tissues, ultimately also causing insulin resistance in the brain [65]. Hyperinsulinemia can further increase inflammation in the brain, and this may contribute to central insulin resistance [65]. Impairment of insulin signaling in the CNS, as a consequence of central insulin resistance, may lead to hyperphagia, weight gain, obesity, and the potentiation of gluconeogenesis by the liver [66]. Thus, obesity is a state in which the negative feedback of insulin in the CNS has become ineffective due to insulin resistance. The CNS resists the regulatory effects of insulin, so that appetite remains uncurbed and weight increases despite adequate energy stores [64].

## 7. The Relationship Between Obesity and Type 2 Diabetes

**Figure 4:**
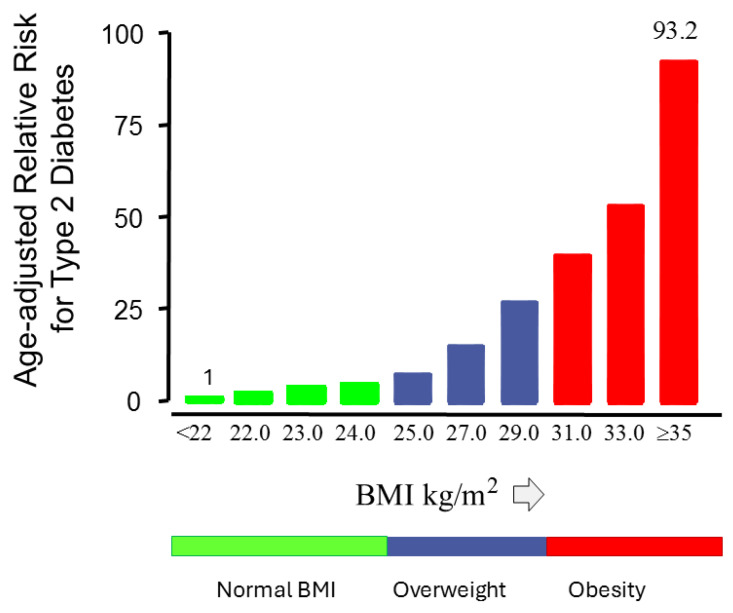
Age-adjusted relative risk for type 2 diabetes and body mass index (BMI). In the Nurses’ Health Study, 114,281 female registered nurses aged 30 to 55 years who did not have diagnosed diabetes mellitus in 1976, were prospectively followed for 18 years. Excess risk for diabetes was found in women with normal BMIs (<25 kg/m^2^) and overweight (BMIs between 25–30 kg/m^2^) at baseline. For example, women with a BMI of 23.0 to 25.0 kg/m^2^ at baseline had a four-fold increased risk for diabetes mellitus compared to women whose body mass index was less than 22.0 kg/m^2^. Thus, in many women, excessive risk for type 2 diabetes was present independent of obesity. Modified from [74].

Mechanisms responsible for the link between obesity and type 2 diabetes are very complex [67]. Obesity has long been associated with insulin resistance and for many years, the prevailing view has been that obesity-induced insulin resistance was the main driver of the worldwide epidemic of the type 2 diabetes mellitus [68]. However, this has been increasingly questioned. Although people with type 2 diabetes, either non-obese or obese, have modestly greater insulin resistance than non-diabetic people, no difference in insulin resistance was found when obese and non-obese people with normal glucose tolerance were compared [69,70]. This suggests that the impact of obesity/BMI on type 2 diabetes is only minor [70]. In further support of this, although obesity has been associated with insulin resistance, only 8% of individual differences in insulin sensitivity can be explained by differences in BMI [71]. Thus, individual variation in insulin sensitivity largely exists independent of the degree of generalized obesity [71]. In the United Kingdom Prospective Diabetes Study (UKPDS), which was initiated in the seventies of the last century, only a minority (36.6%) of the initially included 3834 people with newly diagnosed type 2 diabetes had obesity at baseline (i.e., a BMI greater than or equal to 30 kg/m^2^) [72,73]. In addition, one in three of those, who at baseline were newly diagnosed with type 2 diabetes in the UKPDS, had normal BMIs (i.e., BMIs less than 25 kg/m^2^) [69]. In the Nurses’ Health Study, a prospective study performed from 1976 to 1990, relative risk for type 2 diabetes in the Nurses’ Health Study steadily increased with increasing BMI [74]. However, it was found that women with average body weight at baseline (i.e., a body mass index between 23.0 and 25.0 kg/m^2^), had a four-fold excessive risk for type 2 diabetes compared to women whose body mass index was less than 22.0 kg/m^2^, suggesting that excessive risk for type 2 diabetes is present independent of obesity [74] (Figure 4).

Although BMI is a widely used tool, its use has significant limitations: for example, it is not a reliable indicator of ectopic fat accumulation. Individuals with normal or overweight BMIs can be in possession of significant amounts of ectopic fat depositions [75]. However, the amount of ectopic fat accumulation likely plays an important role in the risk for type 2 diabetes [76] (see also below).

## 8. The Relationship Between Ectopic Fat Storage, Insulin Resistance, and Type 2 Diabetes Mellitus

The portal/visceral hypothesis proposed that particularly an increased visceral/abdominal fat mass leads to increased free fatty acid flux and inhibition of insulin action in the typical insulin sensitive tissues [77]. The portal/visceral hypothesis has been the predominant paradigm for a long time to explain the link between obesity and insulin resistance [77]. However, recent experimental data revealed several flaws in the porta/visceral hypothesis. For example, the relatively small amount of free fatty acids produced by the visceral fat mass is compared to total amount of free fatty acids produced by the subcutaneous fat tissues, not a major contributor of the overall release of free fatty acids to the liver [78,79]. By using isotope dilution/hepatic vein catheterization techniques, Nielsen et al. measured visceral and systemic lipolysis in obese and lean subjects [80]. Although they found that the contribution of visceral lipolysis was proportional to the size of the visceral fat depot, visceral lipolysis was responsible for only 5–10% and 20–25% of total free fat delivery to the liver in lean and obese subjects, respectively [80,81]. In addition, the contribution of visceral lipolysis to systemic (i.e., extrahepatic) free fatty acid availability was very small (generally < 5% [80,81]. Conflicting with the portal/visceral hypothesis, strong associations have been reported between the amount of free fatty acids produced by subcutaneous fat tissues and insulin resistance [82,83]. Since subcutaneous fat tissues do not directly drain into the portal vein, the fatty acid-mediated effects of subcutaneous fat tissues on insulin resistance cannot be explained by the portal/visceral hypothesis [77].

**Figure 5:**
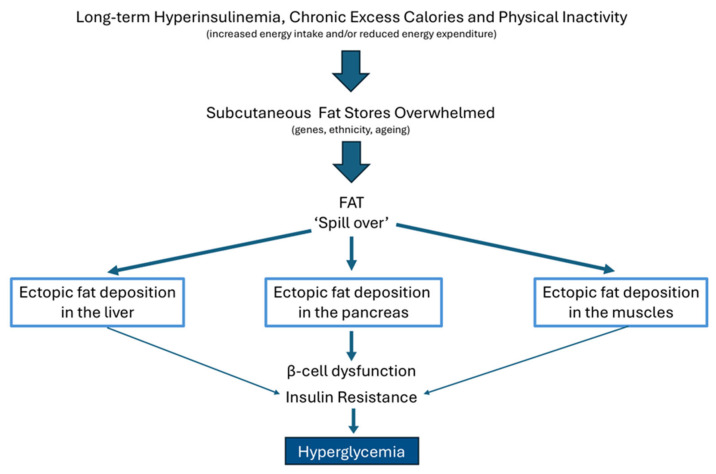
Relation between long-term hyperinsulinemia, chronic intake of excess calories, physical inactivity, subcutaneous fat mass, and the development of ectopic fat depositions in the liver, pancreas, and skeletal muscles. When there is long-term hyperinsulinemia, chronic intake of excess calories, and physical inactivity, the subcutaneous fat tissue can become overpowered (due to an insufficient and limited ability of the subcutaneous fat tissue to proliferate and recruit new fat cells). This may stimulate “spill over” of lipids which results in (ectopic) fat accumulation in the form of triglycerides in non-adipose tissues (such as the liver, pancreas, and skeletal muscles). Deposition of ectopic fat into the liver, pancreas, and skeletal muscles has been hypothesized to explain the established link between insulin resistance, beta cell dysfunction, and type 2 diabetes (see text for details).

Subcutaneous fat tissue is the largest storage site of excess fat in the body [78]. Appropriate energy (fuel) storage is critical in a healthy state: insulin is the main hormone regulating energy storage, in part, by suppressing lipolysis [84]. Normally, subcutaneous fat tissue stores short-term excess energy by lipogenesis in the form of triglycerides, and, upon demand, converts triglycerides by lipolysis into free fatty acids and glycerol to provide energy. However, this process can be overpowered under conditions of chronic overnutrition (long-term intake of excess calories and consumption of the “modern” Western diet) and nutrition-mediated hyperinsulinemia [84] (Figure 5). Limited capacity of subcutaneous fat tissue to expand and store fat, and increased lipolysis, may stimulate lipid spillover, and this may promote fat accumulation in the form of triglycerides in non-adipose tissues (such as the liver, pancreas, and skeletal muscles). Non-adipose tissues normally contain minimal amounts of fat. Accumulation of triglycerides in non-adipose tissues is abnormal and referred to as ectopic fat deposition.

When subcutaneous fat tissue is no longer able to accommodate excess energy (due to an insufficient and limited ability of the subcutaneous fat tissue to proliferate and recruit new fat cells), there will be altered fat partitioning producing a pattern of fat deposition, which shows parallels with (partial) lipodystrophy in humans: excess calories will be stored as ectopic fat (triglycerides) in the abdomen, liver, pancreas, and skeletal muscle [77,78,85]. The ability to accommodate excess fat in the subcutaneous fat tissue varies markedly between individuals because of a combination of genetic and environmental factors [78,86]. It has been postulated that type 2 diabetes will develop when an individual of any BMI can no longer store triglycerides safely in the subcutaneous fat tissue (Personal Fat Threshold hypothesis) [69,86]. From that moment on, excess liver fat accumulation, excess hepatic fat export, and exposure of beta cells to excess lipids will be promoted [69,86].

Deposition of ectopic fat into the liver, pancreas, and skeletal muscles has been hypothesized to explain the established link between insulin resistance, beta cell dysfunction, and type 2 diabetes [77] (Figure 5). Ectopic fat deposition into the liver, pancreas, and skeletal muscles may modulate insulin sensitivity and insulin production [73,74]. Hepatic steatosis, and excess fat in muscles and pancreas, may induce hyperglycemia by overproduction of glucose by the liver, reduction in insulin secretion by the pancreas, and decreased insulin-mediated glucose disposal due to muscle insulin resistance [70] (Figure 5). This trilogy of defects may result in impaired glucose intolerance and type 2 diabetes [70,75].

Several studies demonstrated that the degree of ectopic fat deposition in the liver and skeletal muscle strongly correlates with insulin resistance [77] (see further below). Failure to develop an adequate subcutaneous fat tissue mass, in either humans or mice with lipodystrophy, results in severe insulin resistance and type 2 diabetes [77]. Severe insulin resistance and type 2 diabetes in lipodystrophy is thought to be the direct result of ectopic fat depositions into the liver, skeletal muscle, and pancreas [77]. In patients with lipodystrophy limited expandability of subcutaneous fat tissue exerted a larger effect than the visceral abdominal tissue on the development of insulin resistance and metabolic complications [87]. The obesogenic environment in many Western societies produces in a considerable number of subjects a pattern similar to lipodystrophy: excess lipids are stored as triglyceride in liver and skeletal muscle followed by insulin resistance, glucose intolerance, and type 2 diabetes, suggesting that the adipose tissue mass in these subjects is inadequate to sequester dietary lipid away from the liver, skeletal muscle, and pancreas [77]. Increasing evidence supports that partial “subtle” lipodystrophy may be a factor in the pathogenesis of insulin resistance/metabolic syndrome/type 2 diabetes in the general population. For example, Agrawal et al. identified “lipodystrophy-like phenotypes” in approximately 1 in 8 participants in the UK Biobank cohort and 1 in 20 participants in the Fenland Study cohort [88]. In another population-based study, Lotta et al. found that 53 common genetic variants associated with higher fasting insulin levels were associated with a lipodystrophy-like phenotype (lower levels of gynoid and leg fat mass), higher triglyceride levels, lower HDL cholesterol levels, and type 2 diabetes [89]. Moreover, individuals who carried these 53 common genetic variants showed an impaired capacity to increase hip fat mass and were more likely to develop insulin resistance and type 2 diabetes during chronic overnutrition [90]. Collectively, these data suggest that during chronic overnutrition, partial “subtle” lipodystrophy may be a factor in the pathogenesis of insulin resistance/metabolic syndrome/type 2 diabetes in the general population [91].

## 9. Fat Cell Hypertrophy Correlates with Insulin Resistance

Understanding the pathophysiological molecular mechanisms involved in insulin resistance and exhaustion of the pancreatic beta cells is essential for developing therapeutic strategies that can cure type 2 diabetes. An overactive entero-insular axis has been suggested to play a major role in the pathogenesis of diabetes and obesity [92]. The incretin glucose-dependent insulinotropic polypeptide (GIP) is a hormone secreted by the duodenal K-cells [93]. Early after ingestion of a meal containing high fat and carbohydrates, GIP acutely potentiates glucose-induced insulin secretion [94]. The contribution of GIP to mediating the incretin effect after oral glucose in healthy human subjects is far greater than that of glucagon-like peptide-1 (GLP-1) [95]. Similar results have been found after mixed meals [95]. Endogenous GIP has, therefore, been established as the major physiological incretin hormone [96,97]. GIP in combination with hyperinsulinemia and a slight hyperglycemia, increases glucose uptake and lipogenesis, inhibits lipolysis, and thereby stimulates deposition of triglycerides in subcutaneous fat tissue [98]. This will initially increase subcutaneous fat mass. Expansion of the subcutaneous fat tissue occurs by increasing the pool of new small fat cells (hyperplasia) or by enlargement of pre-existing fat cells (hypertrophy) [99]. Fat cell hyperplasia is the result of a high generation rate of new small fat cells in the subcutaneous fat tissue. Small fat cells in the subcutaneous fat tissue are insulin-sensitive and it is thought that an increased pool of small fat cells in the subcutaneous fat tissue protects from type 2 diabetes [99]. In contrast, fat cell hypertrophy in the subcutaneous fat tissue is associated with a low generation rate of new fat cells [99]. It has been hypothesized that fat cell hypertrophy is a direct consequence of failure of the subcutaneous fat tissue to expand the pool of small fat cells when there is a chronic positive energy balance [100]. When hypertrophic fat cells become too large, they cannot longer effectively store fat. They develop insulin resistance and this lead to lipolysis and fat “spill over”: consequently, fat (triglycerides) is stored in ectopic sites like the liver, pancreas, and skeletal muscles [77]. Subcutaneous fat cell hypertrophy and fat storage in ectopic sites are independent of inflammation and the degree of obesity, and strongly associated with development of insulin resistance and type 2 diabetes [77,100,101].

## 10. Insulin-Mediated Effects on Lipolysis, Glucose Metabolism, and Ectopic Fat Deposition

Insulin plays a key role in the interaction between lipolysis of fat cells, hepatic gluconeogenesis, and regulation of hepatic glucose production [102,103]. By suppressing lipolysis, insulin reduces the release of free fatty acids and glycerol from fat cells. A reduction in free fatty acids and glycerol delivery to the liver decreases hepatic glucose output [104]. On the other hand, when the fat cells develop reduced cellular response to insulin secondary to insulin resistance, lipolysis of the fat cells will increase [84]. Especially enlarged (hypertrophic) fat cells are insulin resistant [105]. Resistance to the antilipolytic effects of insulin leads to the day-long release of free fatty acids by fat cells and elevated plasma free fatty acid levels [105]. When plasma free fatty acids are chronically elevated, this stimulates gluconeogenesis and ectopic fat deposition. As noted earlier, ectopic fat depositions induce hepatic and muscle insulin resistance and impair pancreatic insulin secretion [105]. On the long-term, this may lead to frank diabetes in genetically predisposed individuals [105].

## 11. The Role of Hyperinsulinemia on Tissue-Level Inflammation

Increasing data suggest that diet-driven chronic hyperinsulinemia itself may also cause adipose tissue inflammation [106,107]. As earlier discussed, hyperinsulinemia may lead to hypertrophic and unhealthy fat cells, and this can secondarily attract macrophages and other immune cells [108]. In an animal model, chronic hyperinsulinemia augmented adipose tissue inflammation and insulin resistance by stimulating pro-inflammatory cells (M1 macrophages and natural killer cells), suppressing anti-inflammatory cells (M2 macrophages, eosinophils and regulatory T-cells), and dysregulating metabolic homeostasis [107]. In addition, the hyperinsulinemia-mediated imbalance in M1 and M2 macrophage proportions stimulated iNOS (inducible nitric oxide synthase): arginase-1 imbalance in the adipose tissue [107]. This imbalance promoted extracellular matrix deposition and contributed in this way to insulin resistance in adipose tissue [107]. Thus, in this animal model, modest hyperinsulinemia was sufficient to cause adipose tissue inflammation and to suppress insulin-stimulated de novo lipogenesis in fat cells [109,110]. Interestingly, it was also observed in this animal model that partial reduction in hyperinsulinemia improved insulin sensitivity and attenuated diet-induced abdominal tissue inflammation [107].

## 12. Lipotoxicity May Play a Key Role in the Development of Insulin Resistance in Type 2 Diabetes

Lipotoxicity refers to the harmful effects of excessive lipid accumulation in non-fat tissues. In the literature, it is well established that derangements in lipid metabolism may play an important role in the development of insulin resistance in individuals with type 2 diabetes [111]. As discussed earlier, when, due to the chronic positive energy balance, the maximal expansion capacity of the subcutaneous adipose tissue is reached, dysfunctional fat cells may lead to an excess flow of fatty acids from the adipose tissue and this causes liver and skeletal muscle storage of excess triglycerides beyond their normal storage capacity) (i.e., ectopic fat deposition) [111]. Ectopic fat depositions in liver and skeletal muscles are linked to insulin resistance and type 2 diabetes [112]. The triglycerides in ectopic fat appears to be metabolically inert [113]. However, accumulation of intermediates of lipid metabolism, such as diacylglycerol (DAG) and ceramide, have been proposed as mediators of lipid-induced insulin resistance in these tissues [112] (see also next paragraphs). DAG activates protein kinase C, which eventually impairs insulin signaling, while ceramides inhibit Akt/PKB, further contributing to insulin resistance [114].

Dysfunctional fat cells may also lead to altered secretion of adipokines and cytokines [115]. In response to an increase in fat mass, adipokines are released by either adipocytes or macrophages infiltrating adipose tissue [116]. The term adipokines is used for any substance released by adipose tissue [117]. It has been hypothesized that adipokines in combination with certain cytokines contribute to the development of diseases associated with obesity including insulin resistance, inflammation, hypertension, cardiovascular risk, and metabolic disorders [116].

## 13. Plasma Membrane Sn-1,2-diacylglycerol Plays a Critical Role in Insulin Resistance of Fat Cells

**Figure 6:**
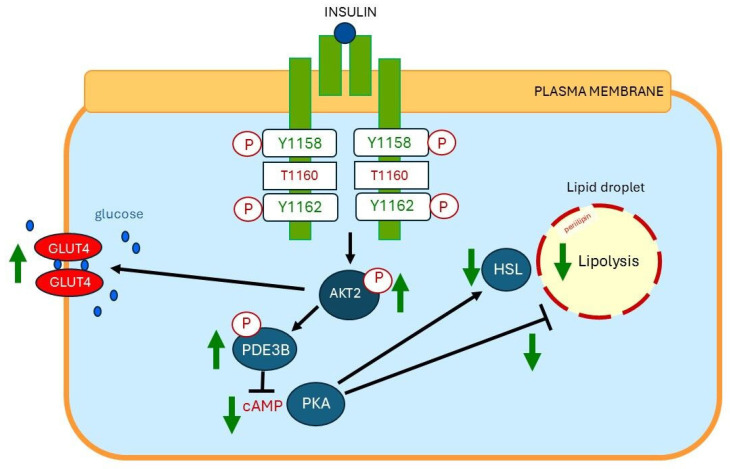

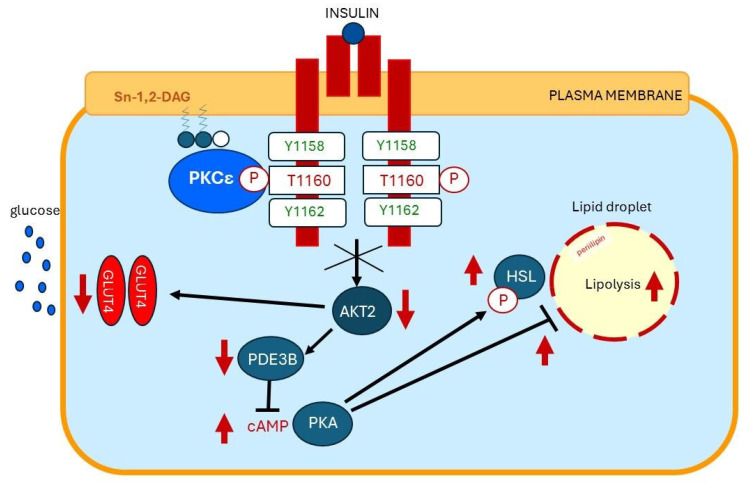
Molecular mechanisms of cellular insulin resistance in the fat cell. (**Top**): Insulin-sensitive insulin receptor (green). Insulin exerts its physiological effects by binding to the insulin receptor on the plasma membrane. Binding of insulin to an insulin-sensitive insulin receptor activates the insulin receptor by stimulating tyrosine residues Y1158 and Y1162 of the beta subunit of the insulin receptor. The activated insulin receptor triggers a signaling cascade that ultimately leads to Akt2 phosphorylation, a key event in regulating glucose uptake and suppressing lipolysis in fat cells. Akt2 phosphorylation promotes the translocation of GLUT4, a glucose transporter, to the cell membrane, enabling glucose uptake by the fat cell. Akt2 activation inhibits lipolysis by inhibiting the activity of hormone-sensitive lipase (HSL), a key enzyme in the breaking down of stored fats. (**Bottom**): Insulin-resistant insulin receptor (red). Overnutrition induces insulin receptor resistance of the fat cell through stimulating plasma membrane (PM) sn-1,2-diacylglycerol (sn-1,2-DAG) accumulation, which promotes protein kinase C-ε (PKCε) activation to impair insulin signaling by phosphorylating insulin receptor threonine T1160. The insulin receptor resistance of the fat cell leads to impaired insulin-mediated glucose uptake and lipolysis suppression of the fat cell. Modified from [84]. Abbreviations Y1158 = tyrosine 1158; Y1162 = tyrosine 1162; T1160 = threonine 1160; P = phosphorylation; GLUT 4 = glucose transporter-4; AKT2 = RAC-beta serine/threonine-protein kinase 2; PDE3B = Phosphodiesterase 3B; cAMP = cyclic AMP; PKA = Protein kinase A; HSL = hormone-sensitive lipase; sn 1,2-DAG = sn-1,2-diacylglycerol; PKCε = Protein kinase C epsilon. Dark blue dots = glucose molecules; ↑ = stimulation: ↓ = inhibition.

Insulin action in subcutaneous fat cells is dysregulated by overnutrition with a high fat diet [84]. Recently Shulman et al. hypothesized that overnutrition induced insulin resistance of the fat cells through sn-1,2-diacylglycerol accumulation in the plasma membrane (PM sn-1,2-DAG) of the fat cells [84]. The accumulation of PM sn-1,2-DAG, in turn, promotes higher protein kinase C-ε (PKCε) activation, which induces phosphorylation of threonine 1160 of the insulin receptor (Insr Thr 1160) (Figure 6). This results in insulin resistance (impaired insulin signaling) of the fat cells [84]. Thus, the plasma membrane sn-1,2-diacylglycerol (PM sn-1, 2 DAG)/PKCε/Insr Thr1160 phosphorylation pathway plays a key role in the pathogenesis of insulin resistance of subcutaneous fat cells [84]. This pathway may be a potential therapeutic target to improve insulin sensitivity of subcutaneous fat cells [84].

## 14. Cause of Increased Diacylglycerol Content in Liver and Skeletal Muscles

Various mechanisms can cause a high net intracellular diacylglycerol (DAG) content in the skeletal muscles and liver [118]. The most common cause is overnutrition [118]. Overnutrition increases the rate of fatty acid delivery to skeletal muscles and liver. DAGs accumulate in skeletal muscle and liver when the rate of fatty acid delivery to these tissues exceeds the rates of intracellular fat oxidation and/or conversion to neutral lipids [118]. Intracellular accumulation of DAGs may lead to muscle and liver insulin resistance by a similar mechanism as described above for the fat cell [118].

## 15. Plasma Membrane Sn-1,2-diacylglycerols Play a Critical Role in Insulin Resistance of Skeletal Muscles and Liver

### 15.1. Insulin Resistance of Skeletal Muscles

Previously, Petersen et al. reported that mitochondrial ATP production in skeletal muscles of young, lean, normoglycemic, insulin-resistant offspring of parents with type 2 diabetes was approximately 30% decreased [119]. They suggested that reductions in mitochondrial content of skeletal muscles were at least in part responsible for the reduced mitochondrial activity [120]. In addition, it was hypothesized that reduction in mitochondrial oxidative phosphorylation caused increased intramyocellular fat content (ectopic fat (triglycerides) within skeletal muscle cells and this was associated with the skeletal muscle insulin resistance [120].

**Figure 7:**
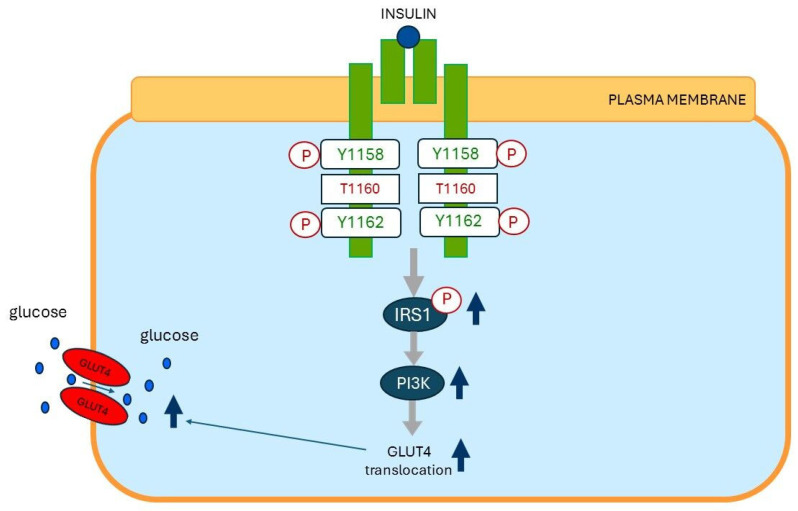

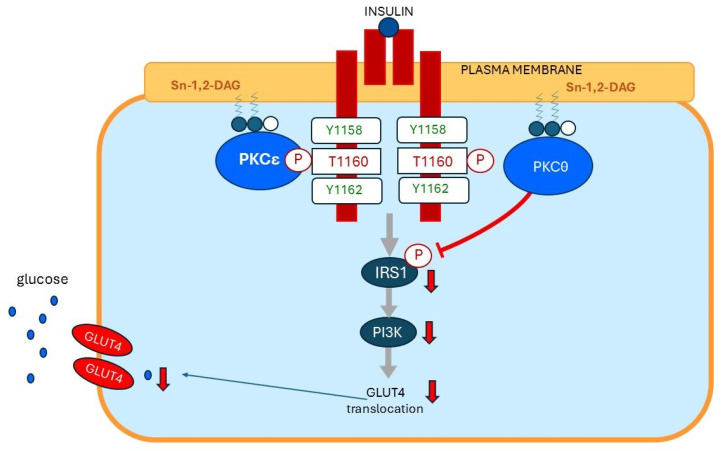
Molecular mechanisms of cellular insulin resistance in the skeletal muscle cell. (**Top**): Insulin-sensitive insulin receptor (green). Insulin exerts its physiological effects by binding to the insulin receptor on the plasma membrane. Binding of insulin to an insulin-sensitive insulin receptor activates the insulin receptor by stimulating tyrosine residues Y1158 and Y1162 of the beta subunit of the insulin receptor. The activated insulin receptor stimulates phosphorylation of IRS1. Phosphorylation of IRS1 leads to stimulation of PI3K, which then activates downstream signaling pathways, including Akt, leading to GLUT4 translocation from intracellular storage compartments to the plasma membrane. This translocation allows GLUT4 to facilitate glucose entry into skeletal muscle cells. (**Bottom**): Insulin-resistant insulin receptor (red). Overnutrition induces insulin receptor resistance of the skeletal muscle cells through stimulating plasma membrane (PM) sn-1,2-diacylglycerol (sn-1,2-DAG) accumulation, which promotes protein kinase C-ε (PKCε) activation to impair insulin signaling by phosphorylating threonine T1160 on the insulin receptor kinase (IRK), which in turn, leads to inhibition of IRK activity, whereas PM sn-1,2-DAG-induced PKCθ activation further contributes to insulin resistance in skeletal muscle by causing reductions in insulin-stimulated IRS-1-associated phosphoinositide 3-kinase (PI3-kinase) activity. This leads to less phosphorylation of IRS1 and PI3K signaling pathways. The insulin resistance of the skeletal muscles impairs the ability of the insulin-sensitive glucose transporter GLUT4 to effectively move glucose from the blood into muscle cells. Modified from (122). Abbreviations Y1158 = tyrosine 1158; Y1162 = tyrosine 1162; T1160 = threonine 1160; P = phosphorylation; IRS1 = insulin receptor substrate-1; PI3K = Phosphoinositide 3-kinase; GLUT 4 = glucose transporter-4; sn 1,2-DAG = sn-1,2-diacylglycerol; PKCε = Protein kinase C epsilon; PKCϴ = Protein kinase C theta. Dark blue dots = glucose molecules; ↑ = stimulation; ↓ = inhibition.

Muscle insulin resistance is a key future of type 2 diabetes and strongly associated with increased intramyocellular lipid content [121]. Lipid infusion in healthy, lean subjects induced acute insulin resistance [121]. The acute induction of muscle insulin resistance resulted in a transient increase in total and cytosolic sn-1,2-diacylglycerol (1,2 DAG) content. The transient increased DAG content temporally caused insulin resistance by increasing protein kinase C-θ (PKC-θ) activation and inhibiting insulin-stimulated IRS-1 tyrosine phosphorylation and AKT2 phosphorylation [121]. Muscle DAG content, PKC-θ activation, and muscle insulin resistance showed a similar pattern in healthy insulin-resistant obese subjects and obese type 2 diabetic subjects [121]. In addition, no associations were observed between insulin resistance and muscle ceramide, acylcarnitine content, or adipocytokines [121]. Increases in plasma membrane sn-1,2-diacylglycerols (PM sn-1, 2 DAGs) in skeletal muscle activate PKC-ε and PKC-θ [122]. PKC-ε activation promotes insulin resistance in skeletal muscles through phosphorylation of threonine 1160 of the insulin receptor kinase (IRK-T 1160), which in turn, leads to inhibition of IRK activity [122] (Figure 7). In addition, PKC-θ activation in skeletal muscle inhibits insulin signaling by phosphorylating IRS1 at Ser1101 [121]. This contributes in skeletal muscles to reduced insulin-stimulated IRS-1-associated phosphoinositide 3-kinase (PI3-kinase) activity [123] (Figure 7). Taken together, all these data suggest a key role for DAG-mediated activation of PKC-ε and PKC-θ in the pathogenesis of insulin resistance in skeletal muscles.

### 15.2. Insulin Resistance of Hepatocytes

**Figure 8:**
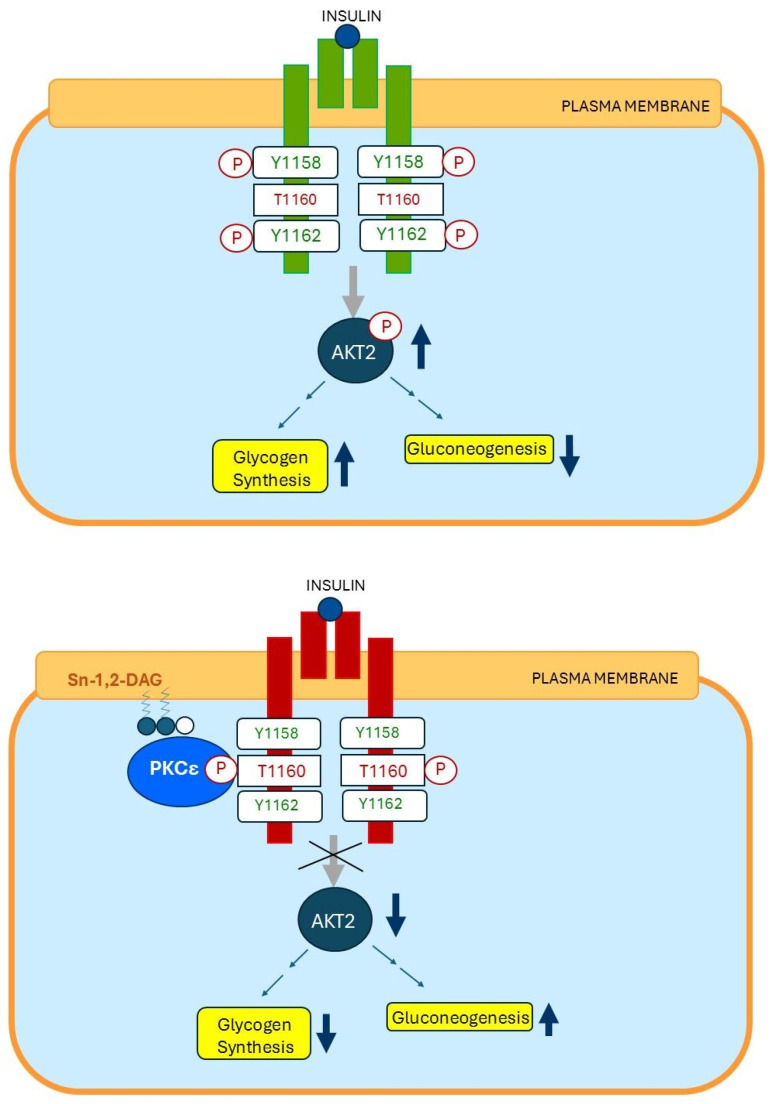
Molecular mechanisms of cellular insulin resistance in the hepatocytes. (**Top**): Insulin-sensitive insulin receptor (green). Insulin exerts its physiological effects by binding to the insulin receptor on the plasma membrane. Binding of insulin to an insulin-sensitive insulin receptor activates the insulin receptor by stimulating tyrosine residues 1158 and 1162 of the beta subunit of the insulin receptor. The activated insulin receptor stimulates activating downstream signaling cascades, notably the AKT2 pathway, which simultaneously stimulates glycogen synthesis and inhibits gluconeogenesis. (**Bottom**): Insulin-resistant insulin receptor(red). Overnutrition induces insulin receptor resistance of the skeletal muscle cells through stimulating plasma membrane (PM) sn-1,2-diacylglycerol (sn-1,2-DAG) accumulation, which promotes protein kinase C-ε (PKCε) activation to impair insulin signaling by phosphorylating threonine 1160 on the insulin receptor kinase (IRK), which in turn, leads to inhibition of IRK activity and downstream signaling. This results in decreased hepatic glycogen synthesis, owing to decreased activation of glycogen synthase, and increased hepatic gluconeogenesis. Modified from (130). Abbreviations Y1158 = tyrosine 1158; Y1162 = tyrosine 1162; T1160 = threonine 1160; P = phosphorylation; AKT2 = RAC-beta serine/threonine-protein kinase 2 sn 1,2-DAG = sn-1,2-diacylglycerol; PKCε = Protein kinase C epsilon. Dark blue dots = glucose molecules. ↑ = stimulation; ↓ = inhibition.

Several studies reported that a fatty liver is not invariably associated with hepatic insulin resistance. This suggests that the accumulation of triglycerides per se is likely not the main factor in causing hepatic insulin resistance [124,125]. DAG and ceramides, both intermediates of fat metabolism when triglycerides are broken down, are the two best-studied putative mediators and leading candidates proposed to be involved in the development of hepatic insulin resistance [126,127,128,129]. Although molecular mechanisms are still debated, Shulman et al. convincingly demonstrated that fatty acid-induced hepatic insulin resistance is primarily caused by plasma membrane-bound PM sn-1,2-DAG accumulation in the plasma membrane of hepatocytes. PM sn-1,2-DAG promotes PKCϵ activation which stimulates insulin receptor kinase-threonine 1160 (IRK-T 1160) phosphorylation, and this results in hepatic insulin receptor resistance [130] (Figure 8). Thus, in this concept, PM sn-1,2-DAG is the key pool of lipids that mediates insulin resistance at the plasma membrane of the hepatocytes.

We hypothesize that DAG-mediated insulin receptor resistance is an adaptation of the cells protecting fat cells, skeletal muscle cells, and hepatocytes against (further) nutrient overload and fuel toxicity.

Although Shulman et al. observed a strong association between intracellular lipid droplets containing DAGs and insulin resistance in hepatocytes, they showed that sequestration of sn-1,2-DAGs in intracellular droplets in hepatocytes does not cause insulin resistance [130,131]. Moreover, no consistent association was observed by Shulman et al. between any ceramide species in any subcellular compartment and hepatic insulin resistance. This is in line with prior studies that reported a dissociation between hepatic ceramide content from hepatic insulin resistance [130,132]. Thus, ceramide likely does not play a major role in hepatic insulin resistance.

## 16. Ectopic Fat and the Pancreas

When, due to chronic overnutrition, fat can no longer be stored in the “safe” subcutaneous fat depots, excess fat is also directed towards the pancreas, where it is deposited as ectopic fat. Pancreatic ectopic fat has been reported in beta cells and exocrine parenchyma [133,134]. Although an adequate supply of free fatty acids in the fasting state is essential for a normal pancreatic beta cell function and insulin secretion, sustained exposure of pancreatic beta cells to elevated free fatty acid concentrations results in increased triglyceride content in pancreatic beta cells [135,136]. Ectopic fat deposition in the pancreas is “lipotoxic”: chronic exposure of human pancreatic beta cells to elevated free fatty acids decreased glucose-stimulated insulin secretion and pancreatic insulin content, and increased triglyceride content, independent from any deleterious effects of hyperglycemia (“glucotoxicity”) [136]. Hyperglycemia by increasing malonyl coenzyme A inhibits carnitine palmitoyltransferase-1, which is a key enzyme in the process of fatty acid oxidation [137]. Consequently, hyperglycemia decreases mitochondrial β-oxidation of free fatty acids, and this may further promote triglyceride content of the pancreas [137]. Pancreatic ectopic fat accumulation is linked to accelerated loss of pancreatic beta-cells [138]. There is emerging evidence that the amount of ectopic fat may be a significant factor in the development and progression of type 2 diabetes [139]. When pancreatic fat content (by proton magnetic resonance spectroscopy) and beta cell function were evaluated and compared between men with and without type 2 diabetes, mean pancreatic fat content was significantly higher in men with type 2 diabetes than in men without type 2 diabetes (20.4% vs. 9.7%) [140]. Moreover, the amount of pancreatic fat correlated negatively with beta-cell function parameters (insulinogenic index adjusted for insulin resistance, early glucose-stimulated insulin secretion, beta-cell glucose sensitivity, and rate sensitivity) [140].

## 17. Correction of Hyperinsulinemia and Prompt Remission of Type 2 Diabetes After Bariatric Surgery

**Figure 9:**
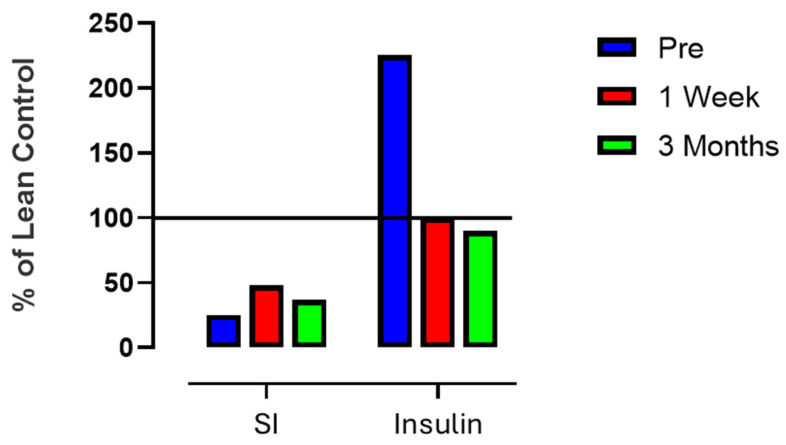
Time course of changes in plasma insulin levels and index of insulin sensitivity within the first 3 months of bariatric surgery (Roux-en-Y gastric bypass). Bariatric surgery corrects hyperinsulinemia within one week of surgery. The normalization of plasma insulin levels after one week coincides with the remission of type 2 diabetes. Before bariatric surgery the index of insulin sensitivity (SI) was low, meaning that the body was less responsive to insulin (i.e., was insulin resistant). The SI remained low and was not normalized 3 months after surgery suggesting that hyperinsulinemia was not secondary, compensating for insulin resistance. Modified from [141].

Type 2 diabetes was very long considered to be a progressive and inexorable disease. However, Pories already provided 30 years ago evidence that gastric bypass surgery can lead to long-term remission of type 2 diabetes [141]. He showed that within one week after bariatric surgery, correction of hyperglycemia was accompanied by the rapid correction of hyperinsulinemia and lowering of fasting insulin levels [141,142] (Figure 9). Moreover, the food stimulated spike of insulin secretion was restored to levels equal to normal lean control subjects [141]. This normalization of fasting insulin occurred independently of any changes of insulin resistance, fasting glucose, and fasting free fatty acids [1] (Figure 9). Changes in insulin levels were observed early and long before there was a significant reduction in body weight and the mass of adipocytes [141,142]. Correction of type 2 diabetes after gastric bypass surgery was durable even though most of these patients remained obese [141]. Correction of type 2 diabetes was less likely in individuals who were older and who had the disease longer [141]. This latter effect was attributed to a lower pancreatic beta cell reserve. Based on these results, Pories proposed that type 2 diabetes starts with a food-mediated diabetogenic signal from the gastrointestinal tract (GIP and/or incretins?) to the pancreatic islets that causes chronic and increasing basal fasting hyperinsulinemia [1]. Therefore, Pories et al. hypothesized that the correction of fasting hyperinsulinemia and remission of type 2 diabetes after gastric bypass surgery is primarily the consequence of this abrupt reduction in food intake [1]. In addition, since normalization of fasting insulin levels after gastric bypass surgery occurred independently from any change in insulin sensitivity, Pories already concluded many years ago that insulin resistance is not the primary reason for hyperinsulinemia but that insulin resistance develops secondary to hyperinsulinemia (probably) to protect against hyperinsulinemia-mediated effects [141].

In a more recent study, obese individuals with normal glucose tolerance or type 2 diabetes were matched for age, weight, and sex, and studied before and 8 weeks after gastric bypass surgery [143]. Eight weeks postoperatively, the observed weight loss and change in total fat mass were similar in both groups [143]. However, the preoperatively increased pancreatic triglyceride content in the group with type 2 diabetes significantly decreased and returned to baseline after bariatric surgery whereas, in contrast, pancreatic triglyceride content in non-diabetic subjects was preoperatively already low (normal) and remained unchanged after bariatric surgery [143]. Simultaneously, first-phase insulin response to a stepped intravenous glucose infusion improved and was normalized in individuals with type 2 diabetes, whereas first-phase insulin response did not change 8 weeks after surgery in individuals with normal glucose tolerance [143]. Thus, despite similar weight loss after bariatric surgery in these two well-matched groups, weight loss specifically decreased excess pancreatic triglyceride content and the first-phase insulin response in the group with type 2 diabetes [143]. These results support the concept that intrapancreatic (ectopic) triglycerides (and/or its metabolites) play a central role in the etiology of type 2 diabetes [143]. In the Swedish Obese Subjects (SOS) study, diabetes remission rate 2 years after bariatric surgery was 72.3%, which agrees with many retrospective and prospective studies [144]. After 15 years of follow-up, the diabetes remission rate decreased to 30.4%, suggesting that short-term remission rates cannot be extrapolated to long-term outcomes [144]. In 2013, it was reported from the SOS study that bariatric surgery reduced the number of new cases of type 2 diabetes in non-diabetic subjects by 96%, 84%, and 78%, after 2, 10, and 15 years, respectively [145]. Thus, in contrast to the decreasing effects of bariatric surgery in time on type 2 diabetes remission, the relatively strong preventive effects of bariatric surgery on the development of type 2 diabetes was, after 15 years, only moderately reduced [146].

## 18. How Does Bariatric Surgery Result in Quick Remission of Type 2 Diabetes?

It has been hypothesized that the introduction of a sudden negative energy balance after bariatric surgery is responsible for the restoration of normal metabolism in type 2 diabetes patients. Due to the sudden energy restriction, liver fat levels fall and normal hepatic insulin sensitivity is restored within days [147]. Simultaneously, plasma glucose levels return to normal. However, insulin receptor sensitivity of muscle and body weight remain abnormal, at least over the weeks and months after bariatric surgery [147]. The sudden negative energy balance after bariatric surgery results in a fall in plasma levels of VLDL1-TG and a lower exposure of beta cells to fatty acids and to locally deposited triglycerides [147,148]. Thus, the removal of fat-induced stress on the pancreatic beta cells postoperatively restores glucose-mediated insulin secretion within some weeks [147].

## 19. Reversal of Type 2 Diabetes After a Very-Low-Calorie-Diet (VLCD)

In patients with poorly controlled type 2 diabetes, moderate weight loss (≈−8 kg) by a hypocaloric very-low-fat-diet (3% fat and ~1200 kcal/day) normalized fasting hyperglycemia and reversed hepatic steatosis [149]. This diet reduced hepatic insulin resistance and normalized basal rates of hepatic glucose production by decreasing gluconeogenesis, and these changes occurred independent of any change in insulin-stimulated peripheral glucose metabolism [149]. This study suggests that mobilization of a relatively small pool of intrahepatic lipid content may normalize dysregulated hepatic glucose metabolism in patients with type 2 diabetes [149]. Moreover, a VLCD did decrease fatty acid oversupply to pancreatic beta cells [149]. In another study, an 8-week VLCD (600 kcal)/day) resulted in a significant reduction in pancreatic fat in individuals with type 2 diabetes [150]. The decrease in pancreatic fat was accompanied by a reversal of type 2 diabetes, normalization of fasting glucose levels, and almost a return to baseline of the first phase insulin response of the pancreas after a glucose load [150]. This study demonstrated that beta cell function of individuals with type 2 diabetes can become normal by acute restriction of dietary energy intake [149]. In the Diabetes Remission Clinical Trial (DIRECT) trial effects of an intervention consisting of withdrawal of antidiabetic and antihypertensive drugs, total diet replacement by a 825–853 kcal per day formula diet for 12–20 weeks, which was followed with stepped food reintroduction (2–8 weeks), and then structured support for weight-loss maintenance was studied in individuals aged 20–65 years who had less than 6 years duration of type 2 diabetes [151]. This intervention resulted after 2 years in a mean weight loss of 7.6 kg and remission of type 2 diabetes in 36% of all participants [151]. Eighty % of participants maintaining a weight loss of more than 15 kg, and 75% of those who maintained over 10 kg weight loss, were after 2 years in remission of type 2 diabetes [151]. Taylor et al. previously demonstrated that >10 kg weight loss resulted in removal of ectopic fat and normalization of fat within liver and pancreas [148,152]. The >10 kg weight loss was associated with durable recovery of beta cell function and normalization of glucose level in the majority [148,152,153]. The above discussed DIRECT study was extended after 2 years, and low-intensity dietary support was then continued in the intervention participants for up to 5 years from baseline [154]. After 5 years, the mean weight loss was 6·1 kg and 13% of the participants were still in remission of type 2 diabetes [154]. The DIRECT study shows that substantial weight loss is associated with sustained remission of type 2 diabetes for 2 years, but after 5 years, there is weight regain and loss of remission of type 2 diabetes [154]. These results have major clinical implications. It suggests that newly diagnosed type 2 diabetes is a potentially reversible condition which in many cases can be corrected for 2 years by diet alone [154]. However, this result shows also that there is need to develop better treatment strategies that can prevent relapses and achieve long-term remission of type 2 diabetes.

## 20. How Does a Very-Low-Calorie-Diet Result in Quick Remission of Type 2 Diabetes?

In an animal model of poorly controlled type 2 diabetes, a 3-day, very-low-calorie diet (VLCD) (one-quarter their typical intake) reversed diabetes before weight loss and this target was reached by multiple mechanisms: [1] A VLCD reduced fasting hyperglycemia by reducing hepatic triglyceride (TAG) and diacylglycerol (DAG) content: this resulted in reduced TAG-DAG-PKCε activation and improved hepatic insulin sensitivity; [2] A VLCD reduced rates of net hepatic glycogenolysis; [3] A VLCD reduced hepatic acetyl-CoA content which led to decreased hepatic gluconeogenesis from pyruvate carboxylase [155]. Note: in this animal model of poorly controlled type 2 diabetes, the VLCD markedly improved hepatic, but not peripheral, insulin sensitivity [155]. In addition, the VLCD-induced reduction in fasting hyperglycemia was observed without any changes in hepatic ceramide content, hepatic inflammatory cytokine concentrations, or plasma concentrations of glucagon, corticosterone, or fibroblast growth factor-21 (FGF-21) [155]. Taken together, all these data show that VLCD reverses type 2 diabetes by pleiotropic mechanisms [155].

## 21. Concluding Remarks

Genetic background, consuming a “modern” Western diet, and long-term overnutrition may all induce hyperinsulinemia. Hyperinsulinemia is an important contributor to systemic insulin resistance and, in certain cases, may (even) precede insulin resistance. When there is chronic fuel overload due to overnutrition, insulin resistance is a physiological defense mechanism of the body protecting insulin-sensitive tissues from fuel overload and fuel toxicity. Subcutaneous fat tissues are the largest storage sites of excess fat in the body. When energy intake is chronically larger than energy expenditure, excess calories will initially be stored in the subcutaneous fat tissues. However, if subcutaneous fat tissues are no longer able to accommodate surplus energy intake, excess fat will be directed toward the liver, pancreas, and skeletal muscles, where it is deposited as ectopic fat.

Overnutrition is thus a major driver of ectopic fat deposition. There is increasing evidence suggesting that ectopic fat deposition directly causes insulin resistance and pancreatic beta cell dysfunction. Overnutrition and ectopic fat increase intracellular diacylglycerol (DAG) accumulation in fat cells, hepatocytes, and skeletal muscles. Translocated DAG into the plasma cell membrane of these three cell types has been proposed as unifying mechanism to explain the development of insulin resistance in these three tissues. Furthermore, ectopic fat accumulation in the pancreas induces beta-cell dysfunction. Bariatric surgery or a very low-calorie diet (VLCD) can quickly reduce ectopic fat from liver and pancreas and decrease intracellular DAG content: this restores hepatic insulin receptor sensitivity, normalizes metabolism, and put type 2 diabetes in many cases in remission. However, over time, there is a considerable loss of remission of type 2 diabetes. Further research is needed to develop new approaches and strategies to achieve long-term remission of type 2 diabetes in all patients.

## Data Availability

Not applicable.
